# Supplementary material for: Are Fear of COVID-19 and Vaccine Hesitancy Associated with COVID-19 Vaccine Uptake? A Population-Based Online Survey in Nigeria
Source: Vaccines (Basel). 2022 Aug 7;10(8):1271. doi: 10.3390/vaccines10081271 (PMC9415607; doi:10.3390/vaccines10081271)
Supplement: Supplementary file 1 [file vaccines-10-01271-s001.zip › Supplementary 1.pdf]

Section B: FEAR OF COVID-19 SCALE

please tick ONE appropriate box

14. I am most afraid of corona

Mark only one oval.

Strongly disagree

Disagree Neutral

Agree

Strongly agree

15. It makes me uncomfortable to think about corona

Mark only one oval.

Strongly disagree

Disagree

Neutral

Agree

Strongly agree

16. My hand become clammy when I think about corona

Mark only one oval.

Strongly disagree

Disagree

Neutral

Agree

Strongly agree

17. I am afraid of losing my life because of corona

Mark only one oval.

- Strongly disagree
- Disagree
- Neutral
- Agree
- Strongly agree

18. When I watch news and stories about corona on social media, I become nervous or anxious

Mark only one oval.

- Strongly disagree
- Disagree
- Neutral
- Agree
- Strongly agree

19. I cannot sleep because I'm worrying about getting corona

Mark only one oval.

- Strongly disagree
- Disagree
- Neutral
- Agree
- Strongly agree

20. My heart races or palpitates when I think about getting corona

Mark only one oval.

- ⬭ Strongly disagree
- ⬭ Disagree
- ⬭ Neutral
- ⬭ Agree
- ⬭ Strongly agree
