# Supplementary material for: Are Fear of COVID-19 and Vaccine Hesitancy Associated with COVID-19 Vaccine Uptake? A Population-Based Online Survey in Nigeria
Source: Vaccines (Basel). 2022 Aug 7;10(8):1271. doi: 10.3390/vaccines10081271 (PMC9415607; doi:10.3390/vaccines10081271)
Supplement: Supplementary file 1 [file vaccines-10-01271-s001.zip › Supplementary 2.pdf]

Section C (last section): VACCINE REFUSAL TOOL and recommendations

please tick ONE appropriate box

21. Are you having doubts about the actual need for the covid vaccine ?

Mark only one oval.

- Yes
- No

22. Are you having concerns about covid vaccine safety ?

Mark only one oval.

- Yes
- No

23. Are you having fear of possible adverse events from covid vaccine?

Mark only one oval.

- Yes No

24. Are you having misconceptions about the safety and efficacy of covid vaccine ?

Mark only one oval.

- Yes
- No

25. Are you having concerns over a possible "immune system overexposure" to covid vaccine ?

Mark only one oval.

- ⬭ Yes
- ⬭ No

26. Did you encounter any past negative experiences with vaccines?

Mark only one oval.

- ⬭ Yes
- ⬭ No

27. In ONE word, describe your fear of covid vaccine if any

____

28. Do you recommend covid vaccine to others?

Mark only one oval.

- ⬭ Yes
- ⬭ No
- ⬭ Maybe
