# Supplementary material for: Are Fear of COVID-19 and Vaccine Hesitancy Associated with COVID-19 Vaccine Uptake? A Population-Based Online Survey in Nigeria
Source: Vaccines (Basel). 2022 Aug 7;10(8):1271. doi: 10.3390/vaccines10081271 (PMC9415607; doi:10.3390/vaccines10081271)
Supplement: Supplementary file 1 [file vaccines-10-01271-s001.zip › Supplementary 3.pdf]

Invitation to participate in a survey on Fear of Covid-19 vaccine in Nigeria

PARTICIPANT INFORMATION AND CONSENT FORM

Dear Respondent,

We sincerely appreciate your intention to participate in our survey. This survey is entirely voluntary and you can skip any question you do not wish to answer.
Please note that the survey is in English language, therefore you must be able to read and understand information in English language to participate.
Please also note that any data provided will be treated with utmost confidentiality and will be used solely for the purpose of this survey research.

What is the survey about?
This survey aims to examine fear of Covid-19 vaccine among Nigerians including its associated factors, people's opinion regarding the vaccine and recommendations. The survey takes about ten (10) minutes to complete.
Please note that our intention for this survey is to write and publish an academic research paper, with a hope to influence public policy about vaccinations.

Who is doing the Research?
We are three (3) PhD students originally from Yobe State, Nigeria.

1. Muhammad Chutiyami, PhD student, Macquarie University, Australia (https://scholar.google.com/citations?user=pcUcGy8AAAAJ&hl=en)
2. Umar Muhammad Bello, PhD student, Hongkong Polytechnic University (https://scholar.google.com/citations?user=FdfnGacAAAAJ&hl=en)
3. Dauda Salihu, PhD student, Hongkong Polytechnic University (https://scholar.google.co.kr/citations?user=dQuhOvsAAAAJ&hl=en)

Why am I being asked to take part and what will I have to do?
We are inviting any Nigerian from the age of 13years and above to participate in this survey.
Participation will require you to respond to the following questions;

- Basic demographic information such as your age, gender, state of origin, educational level etc.

This is to help us examine differences and personal factors affecting Covid-19 vaccine.

- General fear of covid-19 using a "FEAR OF COVID-19 SCALE".
- Fear of taking covid-19 vaccine using a "VACCINE REFUSAL TOOL" and your recommendation toward the vaccine.

Who do I contact for further information?
Should you have any query or further questions, please do not hesitate to contact any of the principal investigators; Muhammad Chutiyami (chutiyamim@gmail.com), Umar Muhammad Bello (umarmbello66@gmail.com), Dauda Salihu (barebaris@gmail.com).

---

* Required

1. If you consent to participate voluntarily in this survey in English language, please click YES * to proceed to the questions.

Mark only one oval.

- Yes
- No
